# Three-Dimensional Point-Cloud Learning for Patient-Specific Deep Brain Stimulation Motor Response Prediction in Parkinson Disease

**DOI:** 10.34133/bmef.0276

**Published:** 2026-09-22

**Authors:** Kaiming Liu, Yinghao Zhu, Ru Wang, Zihao Liu, Gongyi Zhu, Zice Wang, Minyan Ge, Bo Shen, Yimin Sun, Fengtao Liu, Jue Zhao, Narasimha M. Beeraka, Virak Sorn, Haiyin Wang, Vladimir N. Nikolenko, Jianjun Wu, Shumao Xu

**Affiliations:** ^1^ Institute of Science and Technology for Brain-inspired Intelligence (ISTBI), Fudan University, Shanghai 200040, China.; ^2^ Shanghai Health Development Research Center (Shanghai Medical Information Center), Shanghai 201100, China.; ^3^ Department of Neurology and National Research Center for Aging and Medicine & National Center for Neurological Disorders, State Key Laboratory of Medical Neurobiology, Huashan Hospital, Fudan University, Shanghai 200040, China.; ^4^ Department of Human Anatomy and Histology, FSAEI HE I.M. Sechenov First Moscow State Medical University (Sechenov University), Moscow 119991, Russia.; ^5^ Saveetha Institute of Basic Medical Sciences (SIBMS), Saveetha Institute of Medical and Technical Sciences, Saveetha University, Chennai 600077, India.; ^6^ Faculty of Health Sciences & Biotechnology, University of Puthisastra, Phnom Penh 12211, Cambodia.; ^7^ Institute of Clinical Medicine N.V. Sklifosovsky, FSAEI HE I.M. Sechenov First Moscow State Medical University (Sechenov University), Moscow 125009, Russia.; ^8^ Max Planck Institute, Stuttgart 70569, Germany.

## Abstract

**Objective:** To develop a patient-specific 3-dimensional (3D) point-cloud deep learning framework for predicting motor improvement after deep brain stimulation (DBS) in Parkinson’s disease. **Impact Statement:** This study introduces an electric-field-aware point-cloud representation that preserves patient anatomy, electrode geometry, and local electric-field distributions for individualized DBS motor response prediction. **Introduction:** DBS response varies because of complex interactions among electrode location, anatomy, stimulation parameters, and electric field propagation. Existing volume-of-tissue-activated-, atlas-, sweet-spot-, stimulation-map-, connectomic-, and feature-engineered approaches have advanced outcome modeling, but many compress these interactions into thresholded volumes, voxel grids, or global summary features. **Methods:** We integrated preoperative MRI, postoperative computed tomography, electrode reconstruction, tissue conductivity, and finite element electric field simulations into patient-specific 3D point clouds. The screening database contained 561 stimulation records corresponding to 116 initially eligible patients. After exclusion of 37 patients with incomplete or unusable imaging, stimulation, outcome, registration, reconstruction, or point-cloud data, 79 independent patients formed the modeling cohort; one stimulation–outcome record was retained per patient. Patient-specific source point clouds were generated and subsequently downsampled for network processing. **Results:** On the original fixed test set, PointNet++ produced 15 of 24 predictions within ±5 Movement Disorder Society-sponsored revision of the Unified Parkinson’s Disease Rating Scale, Part III (MDS-UPDRS-III) points (62.5%), while the voxelized convolutional neural network produced 8 of 24 (33.3%). Anatomical and stimulation features showed modest predictive value individually, whereas target– and parameter–response analyses revealed substantial interindividual heterogeneity. Given the small test set, these fixed split estimates do not support claims of generalization, superiority, or clinical utility. **Conclusion:** By preserving irregular electrode–tissue geometry and spatially resolved electric field information, the framework provides a spatially faithful basis for individualized DBS motor response prediction and future comparison of candidate stimulation settings.

## Introduction

Personalizing deep brain stimulation (DBS) for Parkinson disease (PD) remains a critical clinical challenge because therapeutic response depends jointly on electrode location, patient-specific anatomy, stimulation parameters, and the spatial distribution of the induced electric field [1–5]. Conventional predictive approaches, including statistical machine learning and volume-of-tissue-activated (VTA) models, often represent these multidimensional interactions using handcrafted summary features, anatomical volumes, or thresholded activation masks [6–10]. Although these representations have provided important clinical and mechanistic insights, they might incompletely retain continuous electric field gradients, directional electrode–tissue interactions, and the spatial relationships between electrode contacts and deep-brain nuclei. DBS targeting the subthalamic nucleus (STN) is an established treatment for advanced PD, with demonstrated benefits for motor symptoms, medication requirements, and functional outcomes [11–17].

The therapeutic effects of DBS nevertheless depend critically on the selection of stimulation amplitude, active contacts, pulse width, and frequency [18–23]. In current practice, these parameters are commonly adjusted through iterative clinical testing to balance motor benefit against stimulation-related adverse effects. This process can be prolonged by symptom fluctuations, delayed responses, and patient fatigue. Directional and segmented electrodes permit more precise steering of the stimulation field, but they also substantially enlarge the parameter space, making exhaustive evaluation of all possible combinations impractical [20,24–26].

To improve the spatial and clinical precision of DBS modeling, several complementary image-guided and data-driven strategies have been developed, including atlas-based electrode localization, patient-specific VTA modeling, probabilistic sweet spots, stimulation–response maps, connectomic analyses, and machine-learning-based outcome prediction [8–10,18–21,27–36]. Atlas- and VTA-based approaches provide clinically useful estimates of electrode position and stimulated tissue, although their results depend on image registration accuracy, tissue conductivity assumptions, and the thresholds used to convert continuous electric fields into discrete activation volumes. Probabilistic sweet spots and stimulation maps identify spatial regions associated with favorable outcomes across patient cohorts, whereas connectomic analyses extend these associations from local anatomy to distributed therapeutic networks. Machine learning approaches further integrate clinical, anatomical, connectivity, and stimulation-related variables to model interindividual differences in treatment response.

**Fig. 1.**
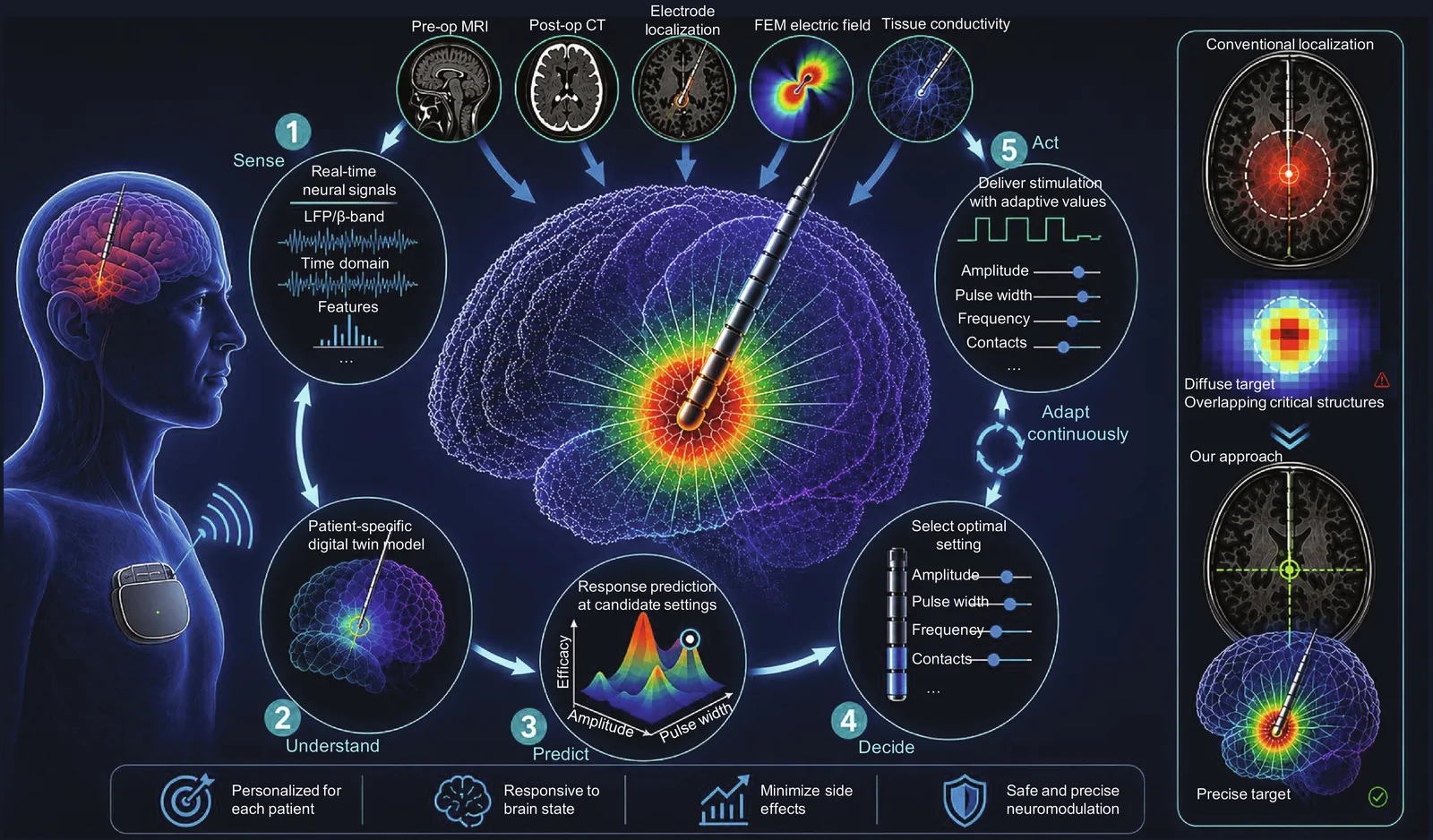
Electric-field-aware 3-dimensional (3D) point-cloud framework for deep brain stimulation (DBS) motor-outcome prediction. Preoperative (Pre-op) MRI, postoperative (Post-op) computed tomography (CT), electrode localization, finite element electric field modeling, and tissue conductivity information are integrated into a patient-specific point-cloud representation for retrospective prediction under observed stimulation settings. FEM, finite element method; LFP, local field potential.

Despite these advances, many existing pipelines transform irregular electrode–tissue geometry into thresholded volumes, regular voxel grids, or global scalar descriptors. These transformations can reduce the spatial resolution of local field gradients and obscure their relationships with patient-specific nuclei, tissue boundaries, and electrode contacts. Because DBS effects arise from spatially heterogeneous interactions between the induced electric field and surrounding neural tissue, a representation that retains per-point anatomical, geometric, and biophysical information can provide a more faithful basis for outcome modeling. Point clouds are well suited to this task because they preserve irregular 3-dimensional (3D) structures while enabling hierarchical learning of both local electrode–tissue interactions and global spatial organization. In this work, we developed a 3D point-cloud deep learning framework that integrates patient-specific anatomy reconstructed from preoperative MRI and postoperative computed tomography (CT), electrode localization using Lead-DBS and PaCER, and finite element electric field simulations incorporating tissue conductivity (Fig. 1). Before network processing, farthest-point sampling selected 512 representative points while maintaining broad spatial coverage; successive set abstraction stages then aggregated local neighborhoods at 128 and 32 points to learn local-to-global features. The model estimated Movement Disorder Society-sponsored revision of the Unified Parkinson’s Disease Rating Scale, Part III (MDS-UPDRS-III; motor examination) improvement for the stimulation configuration observed in each patient, positioning it as an outcome prediction module that can complement electrode localization, field visualization, and clinician-guided programming. The same representation also provides a basis for future within-patient comparison and ranking of candidate settings in prospectively evaluated workflows.

## Results

### Patient cohort and patient-specific DBS point-cloud construction

**Fig. 2.**
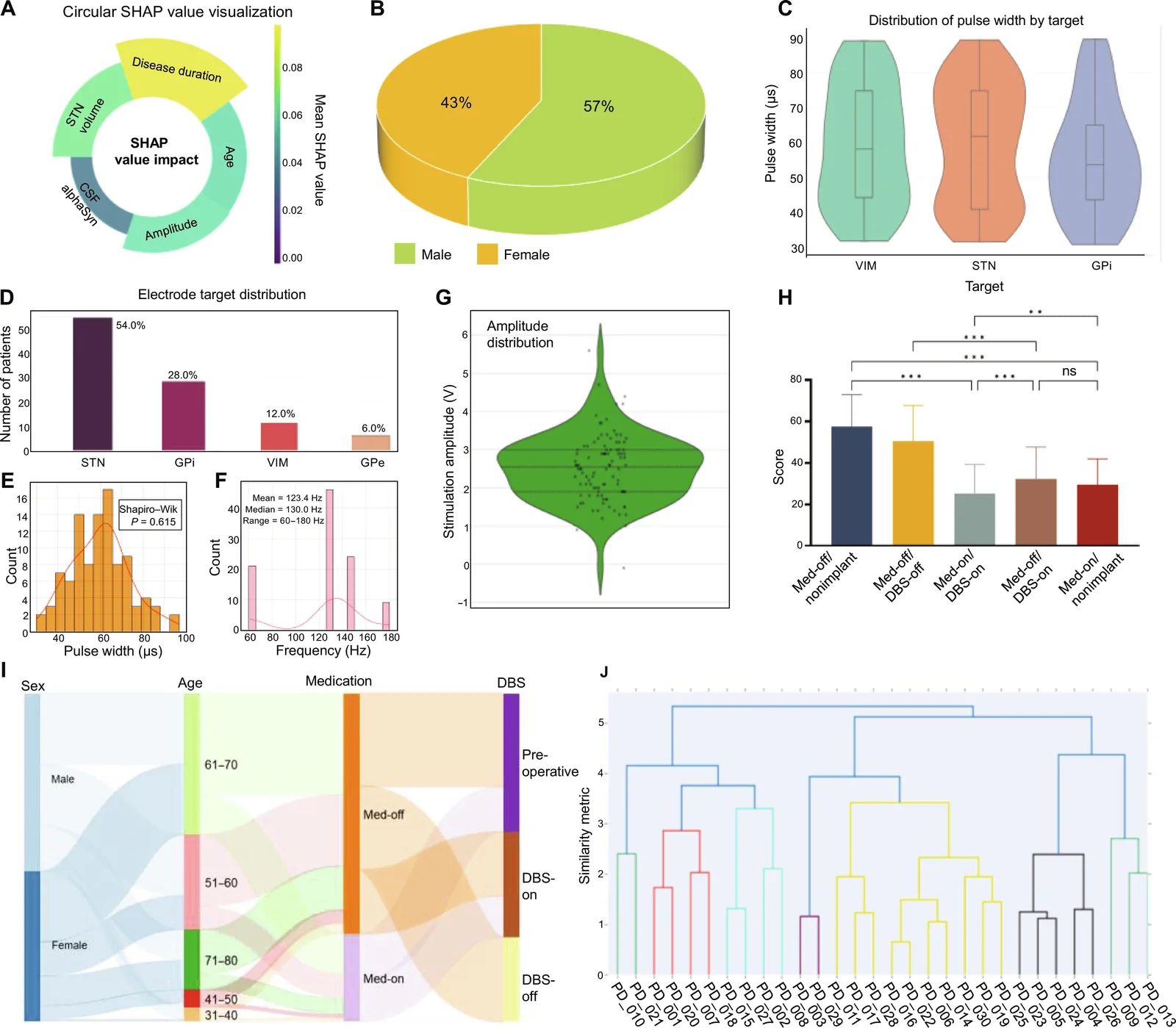
Clinical characteristics and deep brain stimulation (DBS) stimulation parameter patterns of the study cohort. (A) Circular SHapley Additive exPlanations (SHAP) visualization of candidate predictor contributions. (B) Sex distribution. (C) Pulse width distributions by stimulation target. (D) Target distribution. (E to G) Distributions of pulse width, stimulation frequency, and stimulation amplitude, respectively. (H) Movement Disorder Society-sponsored revision of the Unified Parkinson’s Disease Rating Scale, Part III (MDS-UPDRS-III) scores across medication and DBS states. **P < 0.01; ***P < 0.001; ns, not significant (*P* ≥ 0.05). (I) Sankey representation of sex, age group, medication state, and DBS status. (J) Hierarchical clustering of multimodal clinical and stimulation features.

A total of 561 stimulation records were screened. From this pool, 116 patients met the initial clinical and data availability criteria, including confirmed PD diagnosis, implantation with Medtronic 3389S or Pins L301 leads, monopolar stimulation, and availability of preoperative 3-T MRI, postoperative CT, stimulation parameters, and MDS-UPDRS-III scores. Thirty-seven patients were excluded because at least one required imaging, stimulation, outcome, registration, electrode reconstruction, or point-cloud component was incomplete or unusable, leaving 79 independent patients. Mutually exclusive subcategory counts for the 37 exclusions were not available and were not reconstructed. One complete stimulation–outcome record was retained per patient by prioritizing the 6-month postoperative follow-up; when that visit was unavailable, the 12-month record was used, and, otherwise, the record closest to 6 months within the 3- to 18-month window was selected. The study cohort was characterized in terms of baseline clinical features, stimulation target distribution, and observed stimulation parameter patterns (Fig. 2). An exploratory circular SHapley Additive exPlanations (SHAP) analysis summarized the relative contributions of candidate clinical, anatomical, and stimulation-related predictors, highlighting disease duration, age, stimulation amplitude, and STN volume as influential variables within the feature-engineered modeling framework (Fig. 2A). These findings further supported the need to integrate heterogeneous patient-level factors rather than relying on any single clinical or stimulation variable. The cohort had a mean (SD) age of 65.4 (7.8) years, and modest male predominance (Fig. 2B). STN was the predominant target, followed by GPI (globus pallidus internus; internal segment of the globus pallidus), VIM (ventral intermediate; nucleus of the thalamus), and GPe (globus pallidus externus; external segment of the globus pallidus). Stimulation parameters showed target-related structure, including differences in pulse width distributions and recurrent frequency–amplitude combinations (Fig. 2C to G and Figs. S1 to S3). MDS-UPDRS-III scores varied across medication and DBS states, with lower motor scores in active DBS-containing conditions (Fig. 2H). A Sankey diagram summarized relationships among sex, age group, medication state, and DBS status (Fig. 2I), while hierarchical clustering identified patient subgroups with distinct multimodal clinical and stimulation profiles (Fig. 2J and Fig. S4).

**Fig. 3.**
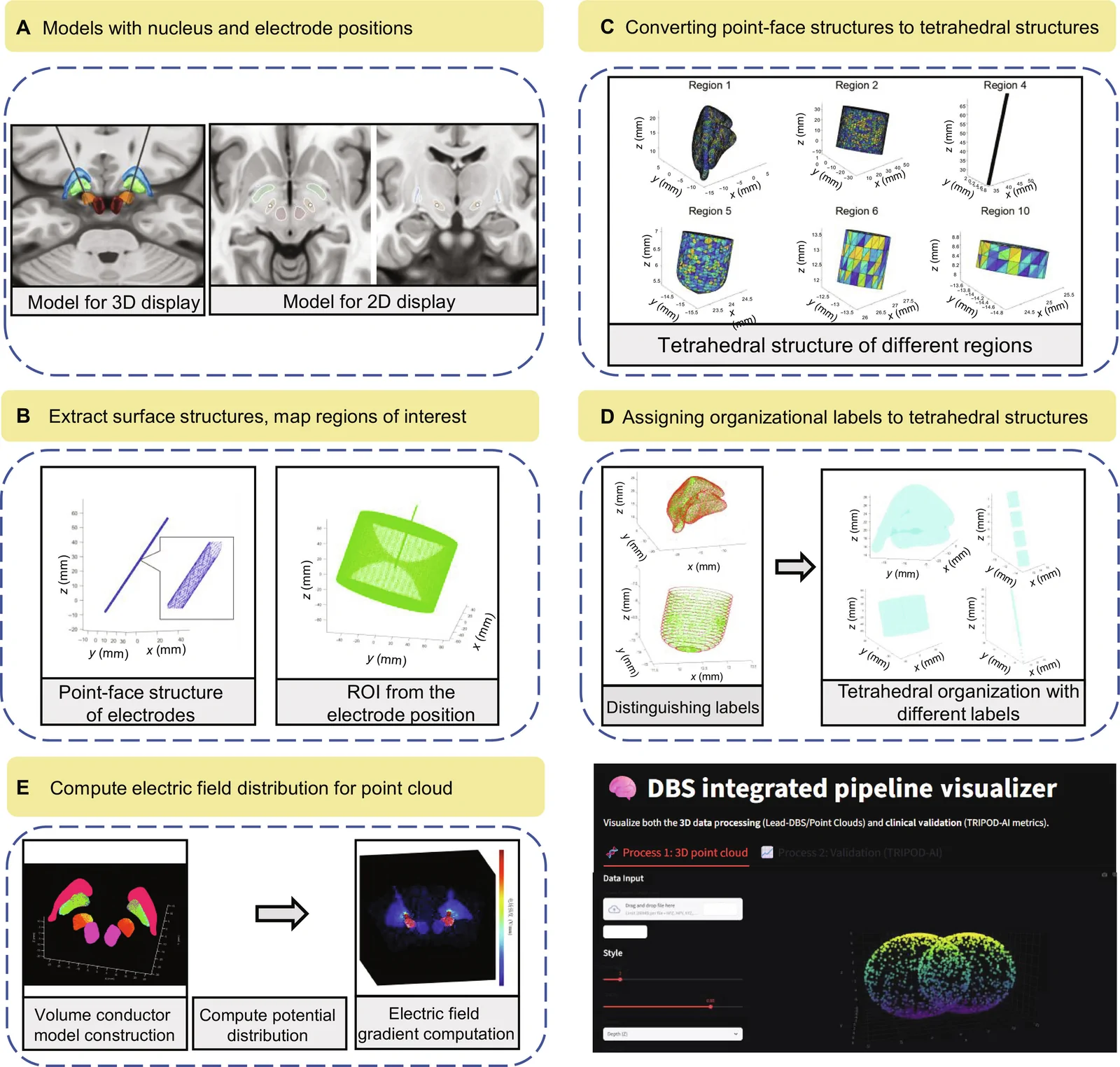
Workflow for constructing the electric-field-aware point-cloud representation. (A) Anatomical and electrode reconstruction. (B) Region of interest (ROI) and surface extraction. (C) Tetrahedral conversion. (D) Tissue and material labeling. (E) Conductivity-weighted electric field computation and spatial fusion.

Electrode reconstruction and nucleus segmentation were performed with Lead-DBS, and postoperative CT was processed with PaCER to reconstruct electrode trajectories and contact locations [13,19,32,33]. The STN and GPi were represented using a subcortical atlas registered to the corresponding anatomical space. Brain shift correction was applied by local linear registration centered on the perielectrode region (Fig. 3A) [36,37]. To generate the biophysical substrate for modeling, we constructed cylindrical regions of interest around the electrode to reduce computational load while preserving relevant tissue context (Fig. 3B) [38]. Surface meshes were converted to tetrahedral meshes with iso2mesh using Delaunay triangulation and Voronoi partitioning (Fig. 3C) [39]. Tetrahedra were labeled according to whether their centroids fell within contacts, insulation, white matter, or nuclei (Fig. 3D) [40], and tissue-specific conductivities were assigned (electrode contacts, 1 × 10^8^ S/m; white matter, 0.14 S/m; gray matter, 0.33 S/m; insulation, 1 × 10^−16^ S/m). Laplace’s equation, *∇* · (*σ∇ϕ*) = 0, was then solved under Dirichlet or Neumann boundary conditions [41,42] to compute electric field distributions, from which field vectors and magnitudes were derived across tissue compartments (Fig. 3E) [43]. Preoperative MRI anatomy was normalized with Statistical Parametric Mapping, version 12 (SPM12)/Advanced Normalization Tools (ANTs), whereas the rigid CT-to-MRI transform, not the nonlinear anatomical warp, was applied to electrode coordinates to avoid distorting the lead trajectory. The Lead-DBS interface used for reconstruction is shown in Fig. S5. The final point-cloud representation therefore encoded anatomy, tissue labels, and local electric field properties in a unified high-resolution space for patient-specific prediction.

### Evaluation of DBS outcome prediction on the fixed internal test set

**Fig. 4.**
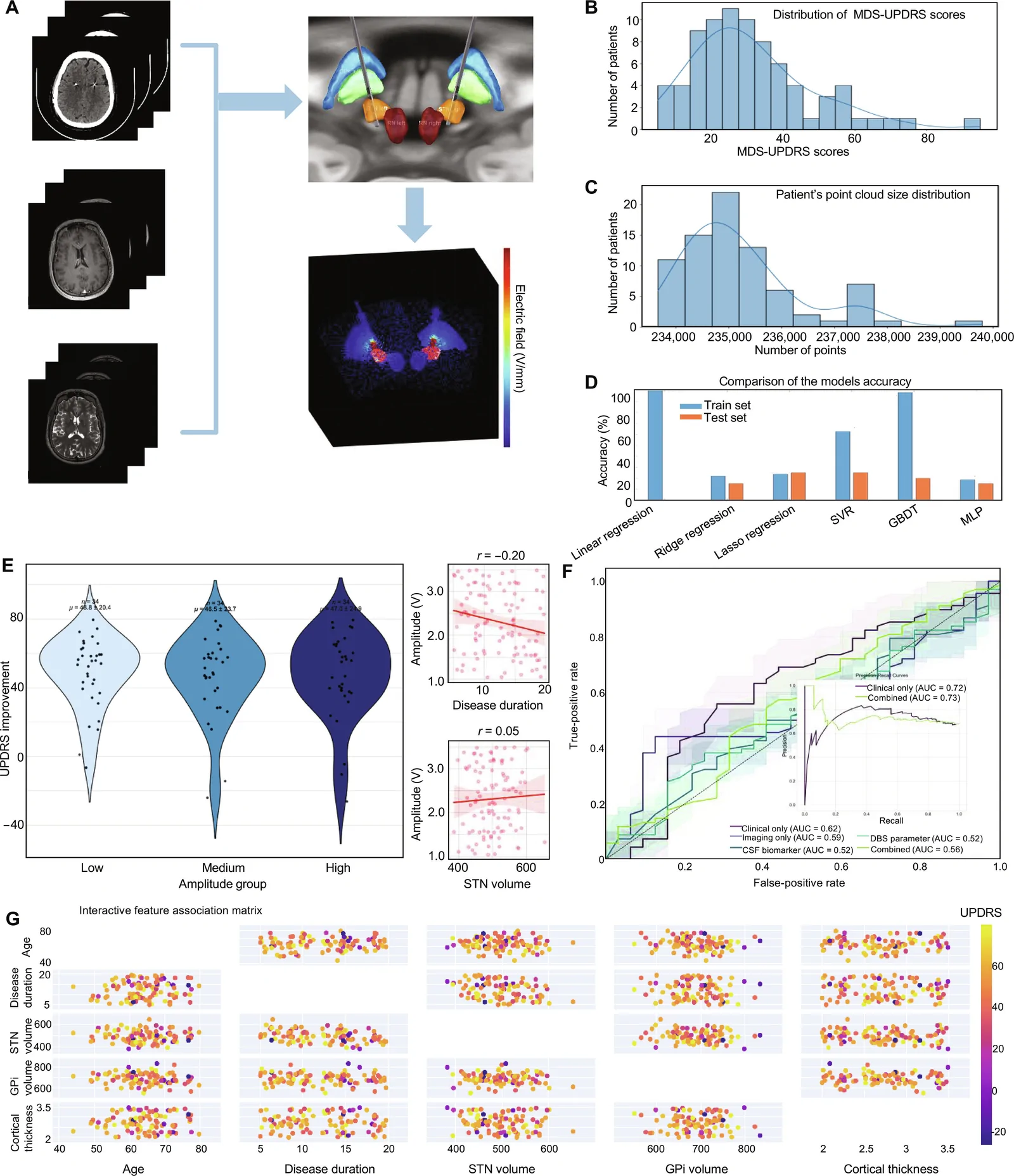
Patient-specific point-cloud construction and fixed split benchmarking. (A) Neuroimaging, electrode reconstruction, and finite element electric field simulation used to generate the source cloud. (B) Movement Disorder Society-sponsored revision of the Unified Parkinson’s Disease Rating Scale, Part III (MDS-UPDRS-III) outcome distribution. (C) Distribution of patient-specific source-cloud sizes before downsampling. (D) Original fixed split tolerance rate display for the models shown. (E) Outcome distributions across amplitude strata. (F) Receiver operating characteristic analyses of feature groups. (G) Association matrix of selected variables. Tolerance rate is the percentage of continuous predictions within ±5 MDS-UPDRS-III points and is not classification accuracy. AUC, area under the curve.

Building on this reconstruction pipeline, we next evaluated the predictive performance of the point-cloud learning framework by preserving electrode–nuclei spatial relationships and local electric field distributions in a unified anatomical–electrophysiological space (Fig. 4A). The clinical outcome distribution showed broad interindividual variability in MDS-UPDRS scores (Fig. 4B), while point-cloud sizes were relatively consistent across patients, supporting comparable geometric complexity for downstream modeling (Fig. 4C).

PointNet++ was benchmarked against linear regression, ridge regression, lasso regression, support vector regression (SVR), gradient boosting decision trees (GBDT), and a multilayer perceptron (MLP) using the same patient-level training and held-out test sets (Fig. 4D and Fig. S6). Feature preprocessing, model selection, and hyperparameter optimization were confined to the training data, and variables derived directly from postoperative outcomes, ΔMDS-UPDRS-III, or responder labels were excluded from the predictor set. The conventional models used a 78D handcrafted feature vector summarizing clinical characteristics, anatomical measurements, electrode localization, stimulation parameters, and electric field statistics. Performance was assessed using tolerance accuracy, defined as the proportion of continuous predictions within ±5 MDS-UPDRS-III points of the observed improvement. Because this measure quantifies the proximity of continuous predictions to the observed score, it was interpreted as an error tolerance metric rather than against a binary chance level. Linear regression and GBDT reached near-complete training tolerance accuracy but substantially lower held-out values; SVR showed intermediate training performance and ~32% test accuracy. Ridge regression and MLP achieved test accuracies of ~21%, whereas lasso regression showed the most balanced training–test profile and the highest conventional held-out accuracy at ~32%. Overall, conventional held-out performance clustered at ~20% to 32%. PointNet++ achieved 62.5% tolerance accuracy, and the supporting voxelized convolutional neural network (CNN) comparison achieved 33.3% (Fig. S7). Ablation analysis further showed lower performance after removal of electric field vectors, electrode geometry, or STN/GPi anatomical labels and after replacement of hierarchical sampling with random sampling, supporting complementary contributions from the integrated spatial representation. The conventional models used the 78D baseline, anatomical, and stimulation-related feature inventory; postoperative outcomes, change scores, and responder labels were excluded from predictors. Missing value imputation and continuous-variable standardization were fitted on the training partition only. Parameter outcome analyses further contextualized the prediction task. Motor improvement varied widely within low-, medium-, and high-amplitude strata (Fig. 4E), and correlations between stimulation amplitude and either disease duration or STN volume were weak. Exploratory receiver operating characteristic analyses of clinical, imaging, stimulation parameter, and combined feature groups showed modest discrimination (Fig. 4F), while the association matrix demonstrated overlapping relationships among age, disease duration, target volumes, cortical thickness, and motor scores (Fig. 4G). These findings indicate that DBS response reflects multivariable interactions rather than any single clinical, anatomical, or stimulation feature.

### Clinical heterogeneity and parameter–response landscapes

**Fig. 5.**
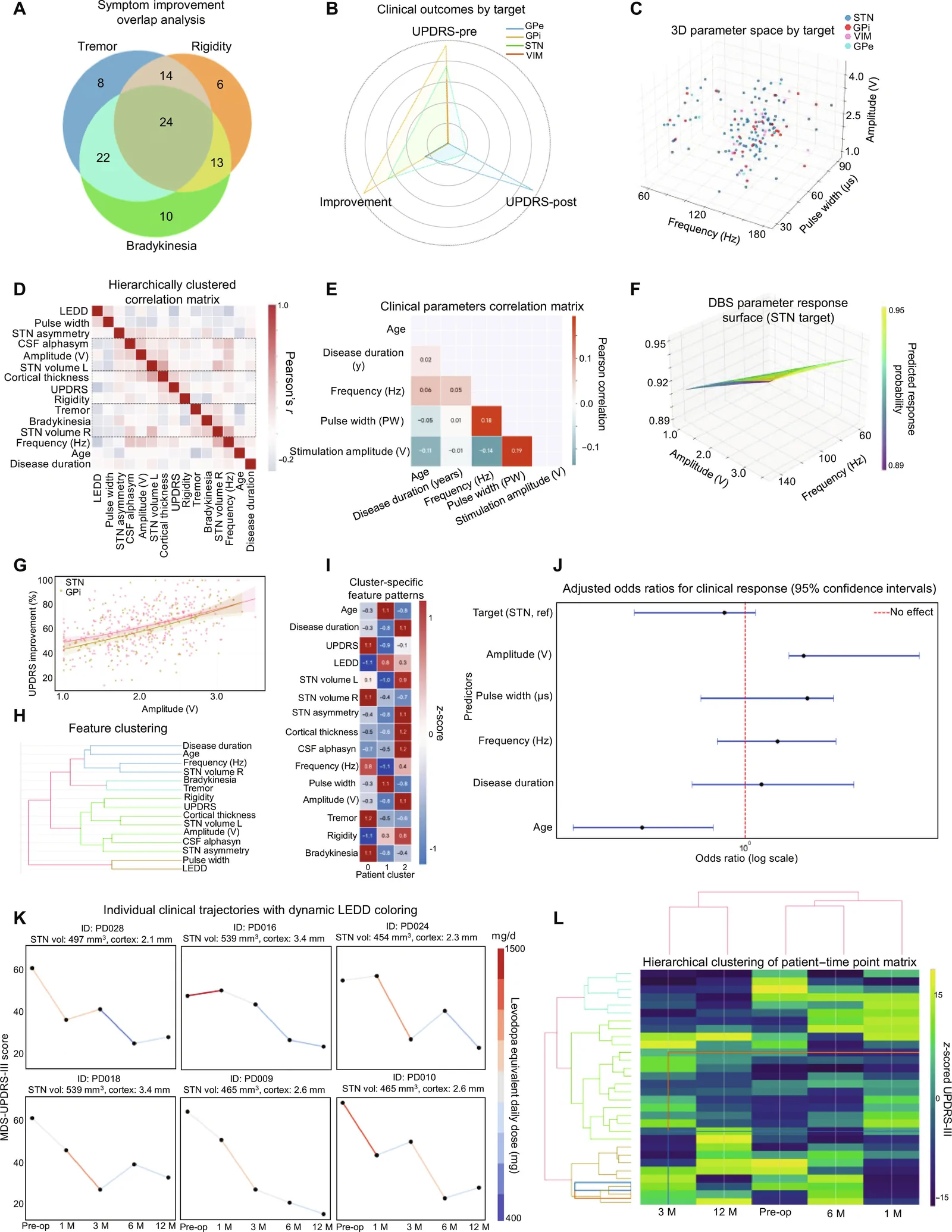
Exploratory clinical heterogeneity and parameter–response summaries. (A to C) Symptom overlap, target-level outcomes, and observed settings. (D to G) Correlation matrices and representative parameter–response displays. (H to J) Feature clustering, subgroup profiles, and adjusted associations. (K and L) Longitudinal trajectories and patient–time point clustering in the available follow-up records. M, month; Pre-op, preoperative assessment.

We next used exploratory clinical, imaging, and stimulation parameter analyses to characterize response heterogeneity (Fig. 5 and Figs. S8 to S12). Symptom-level overlap analysis showed that concurrent improvement in tremor, rigidity, and bradykinesia was common, whereas isolated improvement in a single motor domain was less frequent (Fig. 5A). Outcomes and observed stimulation settings varied across targets (Fig. 5B and C), and correlation matrices showed generally modest relationships among clinical variables, anatomical measures, symptom domains, and stimulation parameters (Fig. 5D and E). The response surface mapped a region of the observed parameter space in which higher predicted responses were concentrated, while substantial overlap across settings and marked interindividual variability emphasized the need for patient-specific modeling (Fig. 5F and G).

Unsupervised clustering resolved structured heterogeneity in the dataset. Feature clustering separated clinical severity, anatomical measures, symptom domains, and stimulation parameters into related groups (Fig. 5H), while cluster-specific *z*-score patterns identified patient subgroups with different combinations of age, disease duration, UPDRS, levodopa equivalent daily dose (LEDD), STN morphology, and symptom response (Fig. 5I). Adjusted odds ratio analysis showed associations of amplitude and age with clinical response, whereas pulse width, frequency, and disease duration had wider confidence intervals (Fig. 5J). Longitudinal trajectories and patient–time point clustering further demonstrated heterogeneous postoperative changes (Fig. 5K and L). These analyses delineated structured variation in symptom trajectories and parameter–response patterns, providing complementary context for patient-specific outcome prediction.

## Discussion

Previous VTA, atlas, probabilistic sweet spot, stimulation map, and connectomic approaches have established clinically relevant relationships between stimulation location and DBS outcomes. Machine learning studies have further shown that clinical, anatomical, connectivity, and stimulation features explain part of the interindividual variability in response. Building on these established approaches, the present framework preserves continuous electric field information and irregular electrode–tissue geometry within a unified learnable representation, providing a complementary strategy for DBS localization and outcome modeling.

The incremental contribution of this study is an electric-field-aware point-cloud representation that retains patient-specific anatomy, electrode geometry, tissue identity, and spatially resolved electric field properties. Unlike thresholded activation masks, global summary variables, or regular voxel grids, the point-cloud formulation preserves local neighborhoods at the electrode–tissue interface and supports hierarchical learning across spatial scales. The ablation analyses and voxelized CNN comparison suggested that multimodal information and the geometry-preserving representation may each contribute to prediction. Because each patient changes the tolerance rate by 4.17 percentage points and the Wilson 95% confidence interval for 15 of 24 is 42.7% to 78.8%, these estimates carry relatively large sampling uncertainty and do not support robust conclusions regarding component-specific effects.

Within the scope of retrospective prediction, the model is directly positioned as a response estimation component within clinician-guided DBS workflows. It predicts motor improvement for an observed stimulation configuration and can be extended to compare multiple candidate configurations for the same patient. This role is complementary to existing localization and visualization platforms, which characterize electrode position and field spread but do not necessarily provide a patient-specific motor-outcome estimate from the same integrated representation. Prospective studies can now evaluate the framework in larger multicenter cohorts using locked patient-level models, temporal or external center testing, and within-patient candidate setting comparisons. Case-level spatial attribution and modality perturbation analyses can further link predictions to influential anatomical and electric field regions. Integration with Lead-DBS, SureTune, Vercise Neural Navigator, or related systems may provide an outcome prediction layer alongside established localization, visualization, and clinician-guided programming functions.

### Limitations

Several limitations define the scope of the present findings. The effective sample comprised 79 independent patients, with only 55 used for fitting in the original split. The layer widths shown imply ~3.6 million trainable parameters under the stated convolution, fully connected, and batch normalization configuration, creating a substantial capacity-to-patient imbalance. The number of points within a patient cloud does not increase the number of independent clinical observations. Training-only augmentation, weight decay, dropout, scheduling, and checkpoint selection can reduce optimization overfitting but do not establish generalization. Repeated leakage-controlled patient-level evaluation and external validation remain necessary.

In summary, this study establishes an electric-field-aware 3D point-cloud framework for predicting motor improvement under observed DBS settings. By preserving patient-specific anatomy, electrode geometry, tissue identity, and spatially resolved electric field properties, PointNet++ achieved 62.5% tolerance accuracy, exceeding the feature-engineered regression models and the voxelized CNN comparison. The framework provides a basis for future evaluation of individualized response estimation and candidate setting comparison. Prospective multicenter validation, leakage-controlled nested reanalysis, and within-patient candidate setting studies are required before clinical decision support claims are made.

## Methods

### Study design and participants

This retrospective cohort study combined multimodal neuroimaging and clinical data to develop a framework for predicting motor outcomes after DBS in PD. A total of 561 stimulation records were screened. The initial eligibility criteria identified 116 patients, including 55 implanted with Pins L301 leads and 61 with Medtronic 3389S leads. Thirty-seven patients were excluded because at least one required imaging, stimulation, outcome, registration, or reconstruction component was incomplete or unusable; the retrospective screening log did not retain mutually exclusive subcategory counts. The final cohort comprised 79 patients. One complete stimulation–outcome record was retained for each patient, so the record distribution in the modeling cohort was one record per patient (median, 1; interquartile range, 1 to 1). All analyzed cases were drawn from the Huashan Hospital clinical cohort.

### Model validation and leakage control

The primary task was continuous prediction of change in MDS-UPDRS-III under the observed stimulation setting. The 79 patients were divided using the original fixed patient-level split into training and internal held-out test sets (Table S1). Predictor preprocessing, model selection, and hyperparameter optimization were performed using only the training data. The held-out test set was reserved for final evaluation. Because one complete modeling record was retained per patient, no patient or stimulation–outcome record was shared between the 2 partitions.

### Evaluation metrics and baseline models

Primary continuous prediction metrics were mean absolute error and root mean square error, with *R*^2^ and correlation treated as complementary measures when available. The ±5-point tolerance rate was the proportion of predictions within ±5 MDS-UPDRS-III points of the observed value and was not classification accuracy or a validated clinical decision threshold. The benchmark comprised linear regression, ridge regression, lasso regression, SVR, GBDT, MLP, a voxelized CNN, and PointNet++, evaluated on the original patient-level split (Figs. S3 and S14). The feature-engineered models received a 78D input vector comprising clinical, anatomical, and stimulation-related features. Postoperative outcomes, change scores, and responder labels were excluded to reduce information leakage risk. Missing values were imputed with training partition medians, and continuous predictors were standardized with training partition parameters. Categorical variables were encoded as binary indicators where applicable. Comparator performance is reported for the original fixed patient-level split using the specified search spaces and source configurations.

### Electric field finite element method simulation and conductivity-weighted Laplace modeling

To compute DBS-induced intracranial electric fields, we solved a quasi-static volume conductor equation with spatially heterogeneous tissue conductivity:

$$\text{∇}\cdot \left[\sigma \left(r\right)\text{∇ϕ}\left(r\right)\right]=0$$ (1)

where *σ*(*r*) denotes the spatially varying tissue conductivity (gray matter, 0.33 S/m; white matter, 0.14 S/m; cerebrospinal fluid [CSF], 1.65 S/m) and *ϕ*(*r*) denotes the electric potential. The electric field was obtained as follows:

$$E\left(r\right)=-\text{∇ϕ}\left(r\right)$$ (2)

Finite element solutions were generated with the COMSOL 5.6 volume conductor workflow. The tetrahedral model used spatially heterogeneous tissue conductivities, and the quasi-static equation *∇* · (*σ∇ϕ*) = 0 was solved to obtain electric potential and the vector field *E* = −*∇ϕ*. The exported fields preserved the spatially heterogeneous conductivity model used in the analysis.

### Multimodal point-cloud generation pipeline

MRI-derived anatomy, PaCER electrode geometry, and finite element electric field vectors were fused into a patient-specific source cloud. CT-to-MRI alignment used a rigid transform for electrode coordinates; nonlinear symmetric normalization (SyN) normalization was limited to anatomical images and was not applied to the lead coordinates. Electric field values were mapped to cloud coordinates with a Gaussian radial basis function. The recorded scale was *ε* = 0.8 mm^−1^, used in the dimensionally consistent kernel *φ*(*r*) = exp[−(*εr*)^2^]. The network input consisted of 7 scalar channels: *x*, *y*, *z*, tissue identity, *E_x_*, *E_y_*, and *E_z_*. Tissue identity was encoded as a single categorical identifier. Electrode contact identity was used during reconstruction and point selection and was not treated as an ordinal continuous input channel [44].

$$\begin{array}{c} E\left(x_{q}\right)=\sum_{i=1}^{N}w_{i}\;\text{exp}\left[-{\left(\epsilon ∥x_{q}-x_{i}{∥}_{2}\right)}^{2}\right] \end{array}$$ (3)

where $x_{q}$ is the query point coordinates, *x_i_* is the finite element simulation nodes [with known *E* from Eq. (2)], *w_i_* is the interpolation weight for the corresponding field component, *r* = ‖$x_{q}-x_{i}$‖ is distance, and *ε* is the inverse-length scale. The recorded *ε* value was 0.8 mm^−1^. Interpolation accuracy was characterized as held-out node validation whenever a fixed subset of nodes was excluded from fitting. The resulting unified point clouds contained ~234,000 to 240,000 points per patient before downsampling, encompassing spatial coordinates, tissue labels (16 anatomical classes), and electric field attributes. Each patient case was stored as a .npz file containing the point-cloud array and outcome variables. The points field contains the point-level input features; y_reg stores the continuous regression target, defined as the change in MDS-UPDRS-III score; and y_cls stores the binary responder label. The y_cls variable was used only as an outcome label and was not included among the PointNet++ input channels.

### Deep learning on point clouds

The reconstructed source cloud was downsampled through hierarchical set abstraction using 512→128→32 points. The PyTorch 1.12 implementation used multiscale grouping (MSG) in the first 2 abstraction stages and global single-scale abstraction in the third. The 7 scalar input channels were *x, y, z*, tissue identity, *E_x_*, *E_y_*, and *E_z_*. The responder label y_cls was not used as a PointNet++ input. For the feature-engineered comparisons, each patient was represented by the 78D vector and modeled using linear regression, ridge regression, lasso regression, SVR, GBDT, and MLP [45,46]. Training-only augmentation comprised rigid rotations of up to ±15° about each axis, a point dropout of 5% to 10%, and an elastic coordinate perturbation recorded with *σ* = 0.5 mm. Rigid rotations were applied to both coordinates and electric field vectors with the same rotation matrix. Because the elastic perturbation was applied to coordinates without recomputing the vector field or applying a local Jacobian, it is treated as a robustness perturbation rather than a physically exact transformation. All augmentation was disabled for evaluation.

$$A=\frac{1}{N}\sum_{i=1}^{N}\left(r_{i}⊗r_{i}\right)$$ (4)

where *r_i_* is the position vector of the *i*th point relative to the centroid and ⨂ refers to the outer product. The eigenvalues of *A* quantify the spatial dispersion of the sampled point geometry along the principal directions, while the corresponding eigenvectors describe the orientations of these directions. While handcrafted descriptors provide interpretability, they can fail to capture higher-order nonlinear dependencies [47]. To solve this, we used PointNet++, a hierarchical deep learning framework tailored for point cloud data. This architecture learns local-to-global geometric features directly from irregular 3D coordinates and associated attributes [48]. To efficiently represent the point cloud, farthest-point sampling selects a subset of points that maximally cover the geometry [49]:

$$\begin{array}{c} {I_{t+1}}^{\left(l\right)}={I_{t}}^{\left(l\right)}\cup \left\{\left|\underset{i\notin {I_{t}}^{\left(l\right)}}{\text{argmax}}\;\right.\underset{k\in {I_{t}}^{\left(l\right)}}{\text{min}}{∥{x_{i}}^{\left(l-1\right)}-{x_{k}}^{\left(l-1\right)}∥}_{2}^{2}\right\} \end{array}$$ (5)

where *I_t_*^(*l*)^ denotes the the set of sampled point indices after *t* iterations. Farthest-point sampling improved spatial coverage of the electrode–tissue geometry. Within each abstraction layer, neighboring points were grouped, relative coordinates and point features were concatenated, shared MLPs were applied, and max pooling generated local features [50]:

$$f_{i}^{\left(l\right)}=\underset{j\in N\left(i\right)}{\text{max}}{\mathrm{MLP}}^{\left(l\right)}\left(\left[f_{j}^{\left(l-1\right)};\;{x_{i}}^{\left(l-1\right)}-{x_{j}}^{\left(l-1\right)}\right]\right)$$ (6)

where $f_{i}^{\left(l\right)}$ is the output feature vector at layer *l* for point *i*, *N*(*i*) denotes its local neighborhood, [·;·] denotes concatenation. The same shared MLP is applied to all neighboring points, and max denotes channel-wise symmetric max pooling. In the MSG stages, neighborhoods were radius based with separate sample caps rather than a single 32-nearest-neighbor rule. The first MSG stage used branch widths [7→64→64→128], [7→128→128→256], and [7→128→196→256]; the second used [640→128→128→256], [640→256→256→512], and [640→256→384→512]. The global stage used [1280→512→768→1024], followed by a regression head [1024→512→256→1] (Fig. S14).

### Training configuration and hyperparameter selection

The recorded objective was $\;L_{\text{total}}=L_{\mathrm{reg}}+0.1L_{\text{corr}}$, with $\left(\frac{1}{N}\right)\text{Σ}\;\text{max}\left(0,\left|y_{i}-{\hat{y}}_{i}\right|-\delta \right)$, *δ* = 5 MDS-UPDRS-III points, and $L_{\text{corr}}=1-r(\hat{y},\;y)$. The ±5-point margin was a modeling tolerance, not an established minimal clinically important difference. Optimization used Adam (initial learning rate 1 × 10^−4^, cosine annealing to 1 × 10^−6^, *β*_1_ = 0.9, *β*_2_ = 0.999), weight decay 1 × 10^−5^, gradient norm clipping at 10, and batch size of 8. Training was capped at 200 epochs. Any checkpoint selection or early-stopping decision was confined to training-derived data; no independent validation cohort was used. Dropout was 0.4 in the fully connected layers. Fixed software seeds were Python = 42, NumPy = 2024, and PyTorch = 42. The layer widths correspond to ~3.6 million trainable parameters under the stated convolutional, fully connected, and affine batch normalization configuration. The sampling sequence, MSG/single-scale grouping (SSG) layout, branch widths, and normalized radii are treated as fixed architectural settings for the reported analysis. The architecture used 512→128→32 hierarchical sampling with farthest-point selection. The first 2 stages used radius-based MSG with 3 scales and sample caps [16, 32, 64] per stage; the third stage used global single-scale abstraction. A single *k* = 32-nearest-neighbor setting was not applied across all MSG branches. Coordinates were centered and scaled to the unit sphere before network input. The dimensionless MSG radii were [0.05, 0.1, 0.2] in the first stage and [0.1, 0.2, 0.4] in the second. Because the physical scale factor was patient specific, these normalized radii do not have one fixed millimeter equivalent and are not converted to approximate physical ranges.

## Data Availability

Anonymized feature vectors and point cloud datasets supporting the findings of this study are available from the corresponding author upon reasonable request and institutional approval. Demonstration code for point-cloud construction, a simplified PointNet++ implementation, and evaluation utilities is available at https://github.com/shumao469/stroke/tree/main/tools/dbs-pointnetpp.

## Supplementary Materials

Supplementary 1 Supplementary Methods Supplementary Text Supplementary Ethical Approval Figs. S1 to S15 Tables S1 to S8
