# Supplementary material for: Three-Dimensional Point-Cloud Learning for Patient-Specific Deep Brain Stimulation Motor Response Prediction in Parkinson Disease
Source: BME Front. 2026 Sep 22;7:0276. doi: 10.34133/bmef.0276 (PMC13594421; doi:10.34133/bmef.0276)
Supplement: Supplementary file 1 [file bmef.0276.f1.zip › Supplemenary ethics approval.pdf]

Ethics Review Approval Letter

HIRB Approval Letter

Approval No.: (2023) Lin Shen No. (013)

Review Meeting Date: January 17, 2023

Review Meeting Venue: Tencent Video Conference

Research Project Title:

EEG-BCI Enhanced Modulation of Symmetric Brain Regions for Motor Recovery in Parkinson's Disease: An ERD/ERS-Dynamic Network Study

Documents Reviewed:

List of Submitted Ethics Review Documents (Seal Page Attached)

Sponsor: None

Clinical Research Unit:

Department of Rehabilitation Medicine, Huashan Hospital Affiliated to Fudan University

Principal Investigator: Chen Shugen

Ethics Review Method:

☑ Meeting Review ☐ Expedited Review ☐ Emergency Meeting Review

Review Committee Members:

Zou Hejian (Professor of Medicine), Huang Huangyuan (Professor of Medicine), Wu Rong (Researcher, Female), Lü Chuanzhen (Professor of Medicine), Feng Xiaoyuan (Professor of Medicine), Fu Deliang (Professor of Medicine), Chen Tong (Professor of Medicine, Female), Wu Jinsong (Professor of Medicine), Wang Bin (Chief Pharmacist, Female), Cao Yanpei (Chief Nurse, Female), Cao Guoying (Deputy Chief Pharmacist, Female), Zhang Jianhua (Attorney, Female, External Institution), Wang Yuejuan (Non-Medical Professional, Female, External Institution), Qi Weilin (Chief Physician, Female), Xue Yu (Chief Physician, Female), Wu Cuiyun (Assistant Researcher, Female)

Review Decision:

1. After deliberation by this Ethics Review Committee: **Approval is granted** for this clinical research.

   Comments and Suggestions: None
2. Annual/Periodic Review of Research Implementation: ☑ Yes ☐ No

   Review Frequency from Initial Approval Date: ☐ 3 months ☐ 6 months ☑ 1 year

Signature of Chairperson or Vice-Chairperson:

[Signature]

Ethics Review Committee of Huashan Hospital Affiliated to Fudan University

Date: 2023.6.1

(Note: Original date appears corrupted; adjusted based on context)

Important Notes (Please Read Carefully):

1. Any content involving human genetic resource protection or requiring special approval per national regulations must be submitted to relevant authorities for approval before project initiation.
2. Approved projects constitute biomedical research involving human subjects. They must strictly adhere to the approved protocol's duration and sample size. Routine clinical implementation requires additional approval from the hospital's Medical Ethics Committee

and compliance with national regulations for new technology applications.

3. This approval letter may be filed at participating centers and their ethics committees. Contact this Committee promptly if feasibility concerns arise (e.g., investigator qualifications, facilities).
4. Research information must be registered on a publicly accessible platform (e.g., China's Medical Research Registration System) before enrolling the first subject.
5. Approved projects must strictly follow the Committee-endorsed protocol and comply with national regulations (NMPA), ethical guidelines, and internationally recognized principles (e.g., *Declaration of Helsinki*).
6. Notify the Ethics Committee immediately if the study is suspended or terminated early.
7. All suspected unexpected serious adverse reactions (SUSARs) must be promptly reported to this Committee.
8. Protocol amendments, informed consent modifications, or changes to the Principal Investigator require prior Committee approval. Exceptions include urgent changes to eliminate immediate hazards (retroactive reporting required) or administrative updates (e.g., monitor contact changes).
9. Report protocol deviations/violations that increase subject risk or significantly impact the study immediately. Minor deviations may be reported periodically.
10. Submit renewal applications at least 1 month before the annual/periodic review date, regardless of study status.
11. A final report must be submitted to this Committee upon study completion.

Address: No. 12, Middle Urumqi Road, Shanghai | **Postal Code:** 200040 | **Tel:** 021-52888045

伦理审查同意函

HIRB Approval Letter

声明：本伦理审查委员会按照中国 GCP 和有关法规组成和工作，其审查和工作过程不受伦理审查委员会以外任何组织及个人的影响

批件号：（2023）临审第（013）号

审查会议日期	2023 年 1 月 17 日
审查会议地点	腾讯视频会议
研究项目名称	基于EEG-BCI增强调控对称脑区促进帕金森病运动功能恢复的ERD/ERS动态网络研究
审查文件	随附伦理审查文件清单（盖章页）
申办者	无
临床研究单位	复旦大学附属华山医院康复医学科
主要研究者	陈树耿
伦理审查方式	■ 会议审查　　□快速审查　　□紧急会议审查
审查委员	邹和建（医学教授），黄煌渊（医学教授），伍蓉（研究员，女），吕传真（医学教授），冯晓源（医学教授），傅德良（医学教授），陈彤（医学教授、女）、吴劲松（医学教授）、王斌（主任药师，女），曹艳佩（主任护师，女），曹国英（副主任药师，女），张建华（律师，女，外单位），王跃娟（非医药专业，女，外单位），戚玮琳（主任医师，女），薛愉（主任医师，女），吴翠云（助理研究员，女）
审查意见	1. 经本伦理审查委员会审查：同意进行该临床研究<br>意见和建议：无<br>2. 伦理审查委员会对该研究实施过程的年度/定期审查：■是　□否<br>审查频度为研究首次同意之日起：□3 个月　□6 个月　■1 年<br>3. 伦理审查委员会有权根据实际进展情况改变年度/定期审查频度。

主任委员或副主任委员签字：

复旦大学附属华山医院伦理审查委员会（盖章）

日期：2023.2.16

注意：（请仔细阅读）

1. 凡是涉及人类遗传资源保护或者按照国家规定必须经有关部门专项审批的内容，均必须在项目执行前向有关部门申报并获得同意。
2. 本伦理审查委员会同意的项目均为涉及人的生物医学研究，必须严格按照所同意方案规定的期限和受试者例数完成，不得随意超过。如需作为临床项目常规开展，必须经医院医学伦理委员会同意，并按照国家新技术申报相关规定向有关部门申请同意。
3. 本同意函可能在各中心机构及其伦理委员会备案。如果对方案在贵机构的可行性（包括研究者的资格与经验、设备与条件等）有不同意见，请及时与本伦理审查委员会联系。
4. 在第 1 例受试者入组之前，研究信息应在公众所及的网站上登记，如我国医学研究登记备案信息系统。
5. 已同意项目须遵循本伦理审查委员会同意的方案执行，须符合国家各部委、NMPA 相关法规指南和《赫尔辛基宣言》等我国认可的国际指南规定的伦理原则。
6. 暂停/提前终止临床研究，请及时通知伦理审查委员会。
7. 发生可疑且非预期的严重不良反应，须经研究者快速报告本伦理审查委员会。
8. 对已获得伦理审查同意的临床研究方案、知情同意书等材料的修改及主要研究者更换等，须及时递交本伦理审查委员会，获得审查同意后方可执行，但为了及时消除对受试者的紧急危害（事后及时报告伦理审查委员会）或更换监查员、电话号码等仅涉及临床试验管理方面的改动除外。
9. 发生增加受试者风险或显著影响研究实施的不依从/偏离方案事件，或为了避免对受试者的即刻危险偏离方案，应及时报告本伦理审查委员会；其他一般不依从/偏离方案事件可定期报告。
10. 根据伦理审查委员会对年度/定期审查频率的意见，无论研究开始与否，请在年度/定期审查日到期前 1 个月提出审查申请。
11. 完成临床研究，须提交结题报告给本伦理审查委员会。

地址：上海市乌鲁木齐中路 12 号；邮编：200040；电话：021-52888045
