# Supplementary material for: Three-Dimensional Point-Cloud Learning for Patient-Specific Deep Brain Stimulation Motor Response Prediction in Parkinson Disease
Source: BME Front. 2026 Sep 22;7:0276. doi: 10.34133/bmef.0276 (PMC13594421; doi:10.34133/bmef.0276)
Supplement: Supplementary file 1 [file bmef.0276.f1.zip › Supporting Informatio-0830.docx]

**Supplementary Information for**

**3D point-cloud learning for patient-specific DBS motor response prediction in Parkinson disease**

*Kaiming Liu,^1,#^ Yinghao Zhu,^1,#^ Ru Wang,^2^ Zihao Liu,^1^ Gongyi Zhu,^1^ Zice Wang,^1^ Minyan Ge,^1^ Bo Shen,^3^ Yimin Sun,^3^ Fengtao Liu,^3^ Jue Zhao,^3^ Narasimha M. Beeraka,^4,5^ Virak Sorn,^6^ Haiyin Wang,^2,*^ Vladimir N. Nikolenko,^7,*^ Jianjun Wu,^3,*^ Shumao Xu^1,8,*^*

^1^Institute of Science and Technology for Brain-inspired Intelligence (ISTBI), Fudan University, Shanghai, 200040, China

^2^Shanghai Health Development Research Center (Shanghai Medical Information Center), Shanghai, 201100, China

^3^Department of Neurology and National Research Center for Aging and Medicine & National Center for Neurological Disorders, State Key Laboratory of Medical Neurobiology, Huashan Hospital, Fudan University, Shanghai, 200040, China

^4^Department of Human Anatomy and Histology, FSAEI HE I.M. Sechenov First Moscow State Medical University (Sechenov University), Moscow, 119991, Russia.

^5^Saveetha Institute of Basic Medical Sciences (SIBMS), Saveetha Institute of Medical and Technical Sciences, Saveetha University, Chennai, 600077, India.

^6^Faculty of Health Sciences & Biotechnology, University of Puthisastra, Phnom Penh, 12211, Cambodia

^7^Institute of Clinical Medicine N.V. Sklifosovsky, FSAEI HE I.M. Sechenov First Moscow State Medical

University (Sechenov University), Moscow, 125009, Russia

^8^Max Planck Institute, Stuttgart 70569, Germany

^*^Correspondence to: [wanghaiyin@shdrc.org](mailto:wanghaiyin@shdrc.org) (H-Y.W.); [vn.nikolenko@yandex.ru](mailto:vn.nikolenko@yandex.ru) (V.N.); [wujianjun@fudan.edu.cn](mailto:wujianjun@fudan.edu.cn) (J.W.); [shumaoxu@fudan.edu.cn](mailto:shumaoxu@fudan.edu.cn) (S.X.)

The Supporting Information includes Supplementary Text, Figs. S1–S15, and Tables S1–S8.

Supplementary methods

Supplementary text

Supplementary Figures 1-15

Tables S1–S8

**Supplement methods**

**1. Support Vector Regression (SVR)**

Insensitive loss function (ε-insensitive loss):

$$L_{0.1}\left( y_{i},f\left( x_{i} \right) \right)=\left\{ \begin{matrix} 0 & \mathrm{if}\left| y_{i}-f\left( x_{i} \right) \right|\leq0.1, \\ \left| y_{i}-f\left( x_{i} \right) \right|-0.1 & \mathrm{if}\left| y_{i}-f\left( x_{i} \right) \right|>0.1. \end{matrix} \right.$$

This means deviations smaller than 0.1 are ignored, while only errors larger than 0.1 are penalized.

Primal optimization problem:

$$\min_{w,b,\xi_{i},\xi_{i}^{*}} \frac{1}{2}\left\| w{\|}^{2}+10\sum_{i=1}^{n} (\xi_{i}+\xi_{i}^{*} \right)$$

subject to

$$\left\{ \begin{matrix} y_{i}-\left( w^{T}\phi\left( x_{i} \right)+b \right)\leq0.1+\xi_{i}, \\ \left( w^{T}\phi\left( x_{i} \right)+b \right)-y_{i}\leq0.1+\xi_{i}^{*}, \\ \xi_{i}\geq0,\xi_{i}^{*}\geq0 \left( i=1,2,\ldots,n \right) \end{matrix} \right.$$

Here:

*w* = model weight vector;

*b* = bias term;

*ϕ(x_i_)* = mapping of input into high-dimensional feature space

ξ_i_, ξ_i_^∗^ = slack variables, allowing tolerance for violations

10 = regularization penalty controlling tradeoff between margin width and error tolerance

RBF (Radial Basis Function) kernel:

$$K\left( x_{i},x_{j} \right)=\exp\left( -\gamma\|x_{i}-x_{j}{\|}^{2} \right)\left( \gamma>0 \right)$$

This kernel transforms data into a higher-dimensional space to handle non-linear relationships.

Dual optimization problem (Lagrangian form):

$$\max_{\alpha_{i},\alpha_{i}^{*}} -\frac{1}{2}\sum_{i=1}^{n} \sum_{j=1}^{n} (\alpha_{i}-\alpha_{i}^{*})(\alpha_{j}-\alpha_{j}^{*})K(x_{i},x_{j})-0.1\sum_{i=1}^{n} (\alpha_{i}+\alpha_{i}^{*})+\sum_{i=1}^{n} y_{i}(\alpha_{i}-\alpha_{i}^{*})$$

Here: α_i_, α_i_^∗^= Lagrange multipliers;

The dual form allows the kernel trick to avoid explicit computation in high-dimensional space.

Prediction function:

$$f(x)=\sum_{i=1}^{n} (\alpha_{i}^{*}-\alpha_{i}^{**})K(x_{i},x)+b$$

This combines support vectors weighted by their multipliers.

**2. Linear regression (LR)**

Model formulation:

$$y=X\beta+\varepsilon$$

Here:

y = response vector

X = design matrix of predictors

β= regression coefficients

ε= error term

Prediction function:

$$\hat{y}_{\mathrm{new}}=X_{\mathrm{new}}\hat{\beta}$$

Loss function (least squares):

$$L(\beta)=\|y-\hat{y}{\|}^{2}=(y-X\beta)^{T}(y-X\beta)$$

Parameter solution (ordinary least squares estimator):

$$\hat{\beta}=(X^{T}X)^{-1}X^{T}y$$

**3. Lasso regression (L1-regularized linear regression)**

The formulation introduces an additional L1 penalty to enforce sparsity in β:

$$\hat{\beta}=\arg\min_{\beta} \left\{ |y-X\beta|^{2}+\lambda|\beta|_{1} \right\}$$

Here: λ= regularization strength

Encourages many coefficients to shrink exactly to zero → feature selection

**4. Gradient Boosted Decision Trees (GBDT)**

An ensemble method that builds trees sequentially. Each new tree fits the residual errors of the previous model:

Base learner: Regression tree

Loss function: Typically squared error for regression or log loss for classification

Update rule:

$$F_{m}\left( x \right)=F_{m-1}\left( x \right)+\eta\cdot h_{m}\left( x \right)$$

where $h_{m}\left( x \right)$ is the new weak learner, and $\eta$ is the learning rate.

**Supplementary Text**

**Section 1: Point cloud data preprocessing pipeline**

Multimodal MRI anatomy, electrode geometry, and finite-element electric-field vectors were integrated into unified patient-specific 3D point clouds through three stages: data acquisition and coregistration, spatial alignment and fusion, and training-only data augmentation.

I. Multimodal data acquisition and co-registration

Anatomical structures: Preoperative 3T MRI (T1/T2-weighted) was segmented using the Lead-DBS toolbox to extract subcortical nuclei (STN, GPi) and surrounding white/gray matter. Segmented volumes were converted to point clouds via surface voxel sampling (0.5 mm resolution), retaining ~150,000 anatomical points per patient.

Electrode geometry: Postoperative CT scans were processed with the PaCER algorithm to reconstruct three-dimensional electrode trajectories and active-contact coordinates. Electrode points (n=12 per contact) were labeled by contact identity and lead-specific geometry. Medtronic 3389S and Pins L301 leads were described by their actual contact configurations without treating all devices as directional leads.

Electric field vectors: Finite-element simulations (COMSOL multiphysics) generated 3D electric field distributions (E-field magnitude, gradient, and directional vectors) at 0.1 mm resolution. Simulations incorporated tissue-specific conductivity (gray matter: 0.33 S/m; white matter: 0.14 S/m) and electrode material properties (1×10^8^ S/m).

II. Spatial alignment and fusion

Coordinate unification: Postoperative CT was coregistered to preoperative MRI with ANTs. A rigid transform was applied to the reconstructed electrode coordinates to preserve the straight lead trajectory; nonlinear SyN normalization was used for anatomical images and was not applied to electrode coordinates. Registration accuracy was not inferred from nominal voxel resolution.

Point-cloud integration: Anatomical points, electrode points, and electric-field vectors were fused using a *k*-d tree nearest-neighbor search (k=32). The reconstructed source cloud contained spatial coordinates, tissue identity, electrode geometry, and electric-field attributes. The seven channels passed to the first set-abstraction layer-^2^ were *x, y, z*, one tissue-identity channel, and E_x_, E_y_, and E_z_. Electric-field magnitude was derived from E_x_, E_y_, and E_z_ for visualization and handcrafted statistical summaries. Electrode-contact identity was retained during reconstruction and sampling but was not encoded as an ordinal continuous network input.

Downsampling and noise reduction: The ~227,500-point reconstructed cloud served as the source representation. Farthest-point sampling selected 512 representative points while maintaining broad spatial coverage, and subsequent PointNet++ set-abstraction stages reduced the representation to 128 and 32 points. Gaussian filtering (σ=0.05 mm) was applied to reduce local simulation noise.

III. Data augmentation

To reduce overfitting, training-only geometric augmentation comprised random rotations (±15° around the x, y, and z axes), elastic deformation (Gaussian kernel, σ=0.5 mm), and point dropout (5%–10% of points). The electric-field vectors (E_x_, E_y_, E_z_) were rotated with the coordinates using the same rotation matrix to maintain physical consistency. Augmentation was applied only to the training cases and was disabled during validation and testing.

PointNet++ architecture: The implementation used MSG in the first two set-abstraction stages and global single-scale abstraction in the third. The sampling sequence was 512→128→32 points. MSG used dimensionless radii [0.05,0.1,0.2] and [0.1,0.2,0.4] with sample caps [16,32,64] in the two stages; these are radius-specific caps, not a single k-nearest-neighbor value. The branch widths and regression head are shown in Fig. S14 and Table S7.

## IV. Quantitative spatial-correspondence validation

Three spatial error sources were considered separately: CT-to-MRI registration, RBF field interpolation, and brain-shift correction. Appropriate validation would require independent cross-modal landmarks or surfaces for registration, held-out FEM nodes for interpolation, and a defined landmark or surface reference for residual brain-shift error.

**Section 2: Mechanistic mapping and comparative validation**

Additional analyses delineated the mechanistic and translational scope of PointNet++. Symptom-specific and connectomic mapping provide natural extensions for relating local electric-field patterns to tremor, rigidity, bradykinesia, and distributed motor pathways. Candidate-setting ranking represents a further extension in which the same patient-specific representation can be used to compare multiple stimulation configurations. The current quantitative model focuses on motor-outcome prediction under observed settings and provides the computational basis for these subsequent analyses. The original validation framework used a fixed patient-level training-test split. The cohort derivation, record distribution, and split design are summarized in Table S1.

I. Data splitting and holdout evaluation

Patient-level split: The final modeling cohort included 79 independent patients. The 561 stimulation records were used for screening and were not treated as independent modeling samples. One stimulation–outcome record was retained per patient by prioritizing the 6-month follow-up, then the 12-month follow-up, and otherwise the visit closest to 6 months within the 3- to 18-month window. The original split assigned 55 patients to training and 24 to an internal held-out test set. All patients were treated at Huashan Hospital. No repeated nested cross-validation or external-center validation result is reported.

II. Performance metrics

Performance metrics: MAE and RMSE are the primary continuous-prediction metrics, with R² and correlation treated as complementary measures when available. The ±5-point tolerance rate is the percentage of continuous predictions within ±5 MDS-UPDRS-III points and is not classification accuracy or a validated clinical threshold. On the original fixed internal test set, PointNet++ produced 15/24 predictions within tolerance (62.5%); the Wilson 95% confidence interval is 42.7%–78.8%. No repeated nested cross-validation performance values are reported.

$\text{L}_{\text{reg}}\text{=}\frac{\text{1}}{\text{N}}\sum_{\text{i}\text{=1}}^{\text{N}} \max\left( \text{0, }\left| \hat{\text{y}_{\text{i}}}\text{-}\text{y}_{\text{i}} \right|\text{-}\text{δ} \right)$

where *N* denotes the total number of training samples, corresponding either to individual patients or specific stimulation configurations included in one training epoch. Each input is indexed by *i*∈[1,*N*], representing a single unified DBS point cloud instance. The model predicts an improvement score $\hat{\text{y}_{\text{i}}}$ from the integrated 3D representation (anatomy + electrode geometry + E-field), while $\text{y}_{\text{i}}$ denotes the clinically observed MDS-UPDRS-III outcome used as ground-truth reference. A tolerance parameter $\text{δ}$ was applied during optimization such that errors smaller than δ=5 UPDRS points did not contribute to the tolerance component of the loss. The ±5-point margin was a predefined modeling tolerance rather than an established minimal clinically important difference. Under this loss, only discrepancies>δ contributed to the regression penalty, preventing the network from overfitting to small score differences within the predefined modeling margin. A Pearson correlation metric was used to evaluate correspondence between predicted and actual postoperative outcomes. Correlation penalty:

$$\text{L}_{\text{corr}}\text{=1-}\text{r}\left( \hat{\text{y}}\text{,}\text{y} \right)$$

where the Pearson correlation across samples quantifies how well the model tracks relative clinical improvement levels. Correlation was treated as a supplementary ranking measure rather than a replacement for absolute-error metrics. Correctly ranking likely responders does not establish that the model can select an optimal stimulation setting for an individual patient.

The network was trained by minimizing a composite objective *L*_total_, updated using backpropagation at every iteration.

$\text{L}_{\text{total}}\text{=}\text{L}_{\text{reg}}\text{+0.1×}\text{L}_{\text{corr}}$

where $\text{L}_{\text{reg}}$ penalized inaccurate estimations of postoperative UPDRS improvement, incorporating the tolerance margin described earlier to ignore clinically negligible deviations; $\text{L}_{\text{corr}}$ enforced monotonic agreement between predicted and actual outcomes, ensuring that the model captured responder ranking even if absolute values were imperfect. A weighting factor of 0.1 was applied to the correlation component, providing stability and preventing it from overwhelming the regression objective.

The Adam optimizer was used with an initial learning rate of 1×10^-4^, adjusted by a cosine annealing scheduler with a minimum rate of 1×10^−6^. Momentum parameters were set to β_1_=0.9 and β_2_=0.999, and an L_2_ weight decay of 1×10^−5^ was applied for regularization. To enhance training stability, gradient clipping with a maximum norm of 10 was employed to prevent exploding gradients during backpropagation. Batch size was 8, training was capped at 200 epochs, and dropout was 0.4 in the fully connected layers. Any checkpoint selection was restricted to training-derived data. The architecture corresponds to ~3.6 million trainable parameters. For transparent evaluation, all models were compared on the original patient-level split. The conventional models used the 78-dimensional feature inventory in Table S5; postoperative outcomes, change scores, and responder labels were excluded. Imputation and scaling parameters were fitted on the training partition.

## III. Baseline preprocessing and hyperparameter optimization

A confirmatory comparison would require a single leakage-controlled nested evaluation protocol in which preprocessing and hyperparameter selection are fitted only within each outer-training partition.

$\text{Accuracy=}\frac{\text{1}}{\text{N}_{\text{test}}}\sum_{\text{k}\text{=1}}^{\text{N}_{\text{test}}} \text{I}\left( \left| \text{y}_{\text{k}}\text{-}\hat{\text{y}_{\text{k}}} \right|\text{≤}\text{δ} \right)$

where I(⋅) refers to an indicator function that returns 1 when the prediction error lies within ±5 points (*δ*=5) of the ground-truth MDS-UPDRS-III motor score, and 0 otherwise. This definition operationalized the original ±5-point tolerance used in the analysis and was not interpreted as a validated clinical decision threshold. The model was optimized using the Adam algorithm, which adjusts learning rates dynamically based on estimates of the gradients’ first and second moments:

$$\text{θ}_{\text{t}}\text{=}\text{θ}_{\text{t}\text{-1}}\text{-}\text{α}\frac{\hat{\text{m}_{\text{t}}}}{\sqrt{\hat{\text{v}_{\text{t}}}}\text{+ϵ}}$$

where $\hat{\text{m}_{\text{t}}}$ and $\hat{\text{v}_{\text{t}}}$ denote the bias-corrected first and second moments of the gradient, ensuring stability during training. Default parameters were adopted (β_1_=0.9, β_2_=0.999, ϵ=10^-8^), which have been empirically validated across a wide range of deep learning tasks, including medical imaging.

**Supplementary Figures**

**Fig. S1.** Stimulation-parameter combinations across targets in the screened source records. Points represent available stimulation records, with color encoding pulse width. The plots show target-specific distributions of frequency, amplitude, and pulse width across the source data.

**Fig. S2.** Demographic and stimulation-parameter distributions in the screened source records, summarizing age, disease duration, amplitude, pulse width, stimulation frequency, and the interval from surgery to device activation. Repeated records from the same patient can contribute repeated demographic values.

**Fig. S3.** Joint distributions of stimulation parameters in the screened source records. Diagonal panels show univariate kernel-density estimates, and off-diagonal panels show bivariate relationships among frequency, pulse width, and amplitude. The distributions reveal recurrent parameter combinations within the observed clinical data.

**Fig. S4.** Patient subtyping by multimodal features. Hierarchical clustering summarizes variation in clinical, anatomical, symptom, and stimulation variables across the final cohort and identifies groups with distinct multimodal profiles.

**Fig. S5.** Lead-DBS interface used for image registration, electrode localization, and anatomical reconstruction.

**Fig. S6. Feature-engineered regression models and fixed-split predictions.** (a) GBDT, (b) SVR, and (c) MLP. Scatter plots show predicted versus observed motor improvement; tolerance rate is the percentage of predictions within ±5 MDS-UPDRS-III points.

**Fig. S7. Supporting fixed-split model and parameter-space comparisons.** The displayed PointNet++ and voxelized-CNN tolerance rates are 62.5% and 33.3%, respectively, on the original held-out split.

**Fig. S8**. **Associations among stimulation parameters, clinical variables, and model attributions**.

**Fig. S9.** **Three-dimensional distribution of observed frequency, pulse width, and amplitude values, color-coded by age.** It illustrates how observed stimulation configurations are distributed across the cohort and highlights the heterogeneity of parameter combinations used in clinical practice.

**Fig. S10. Bivariate and univariate distributions of observed stimulation parameters**. Contours, marginal densities, and scatter distributions summarize the structure and overlap of frequency, pulse width, and amplitude values in the source data.

**Fig. S11. Longitudinal overlap of tremor and rigidity improvement at 3, 6, and 12 months.** The Venn diagrams summarize symptom-domain changes in the available follow-up records.

**Fig. S12. Correlation matrix of selected multimodal variables.** Spearman coefficients summarize pairwise associations among clinical, anatomical, and stimulation features.

**Fig. S13.** **Feature-engineering pipeline used for the conventional regression baselines.**

**Fig. S14. PointNet++ architecture used for DBS motor-outcome prediction.** Seven scalar input channels enter MSG1, whose three branches output 128+256+256=640 channels. MSG2 outputs 256+512+512=1280 channels. Global abstraction then applies 1280→512→768→1024 transformations, followed by a 1024→512→256→1 regression head with batch normalization and dropout in the first two fully connected layers. The declared widths imply ~3.6 million trainable parameters; 768 is an intermediate global-stage width, not the stage output. MSG radii and sample caps are listed in Table S7.

**Fig. S15. Exploratory case-level interpretation, modality perturbation, calibration, and decision-curve displays.** (a–c) Representative cases show the observed and predicted MDS-UPDRS-III change, absolute residual, electrode/anatomy rendering, electric-field magnitude, and post-hoc pointwise attribution. (d) Perturbation displays compare the full-model prediction with changes after suppressing electric-field, tissue-label, or electrode-geometry information.

**Table S1.** Cohort derivation and patient-level training-test split

| **Item** | **Value** | **Clarification** |
| --- | --- | --- |
| Screened stimulation records | 561 | Screening pool; not treated as independent modeling samples |
| Initially eligible patients | 116 | Met clinical and data-availability criteria |
| Excluded before point-cloud modeling | 37 | 37 patients; mutually exclusive subcategory counts were not retained |
| Final modeling patients | 79 | Independent clinical cases |
| Training set | 55 patients | 70% patient-level partition |
| Held-out test set | 24 patients | 30% internal holdout |
| Patient-level separation | Confirmed | No patient or modeling record appeared in both sets |
| External-center test set | No | None; all patients from Huashan Hospital |
| Holdout test used | Yes | Used for final reported tolerance rate |
| Eligible lead types | Pins L301: n=55; Medtronic 3389S: n=61 | Counts refer to the 116 initially eligible patients |
| Final-cohort targets | STN: n=43; GPi: n=22; VIM: n=9; GPe: n=5 | 79-patient target distribution |

**Table S2.** Computational pipeline specifications for PointNet++ framework

| **Component** | **Parameters/methods** | **Techniques** | **Validation metrics** |
| --- | --- | --- | --- |
| **Imaging & reconstruction** | | | |
| MRI acquisition | GE 3T  United Imaging 3T  Philips 3T, | - | Location of STN, GPi, and other nuclei |
| Electrode reconstruction | PaCER algorithm | trajectory accuracy with high precision | The segmentation of electrodes and tissues |
| Finite element modeling | | | |
| Volume conductor | COMSOL 5.6 volume-conductor workflow | Tetrahedral mesh; imported anatomical/material labels | - |
| Tissue conductivity (S/m) | Gray matter: 0.33; White matter: 0.14 | Electrode: 1×10^8^; Insulator: 1×10^-16^ | - |
| Field simulation | ∇·(σ∇ϕ)=0 with Dirichlet/Neumann BCs | High-precision resolution vectors | - |
| **Point cloud generation** | | | |
| Data fusion | K-D tree + RBF interpolation | Fuse the nucleus electrode points with the electric field distribution points | - |
| Spatial attributes | Coordinates, tissue IDs, E-field vectors | ~227,500 points per patient before sampling | Distribution shown in Fig. 4c |
| CT-to-MRI registration | Rigid CT-to-MRI electrode transform; SyN for anatomy only | ANTs 2.3.4 | Independent quantitative TRE not re-estimated |
| RBF field interpolation | Gaussian RBF; held-out-node validation | ε=0.8 mm^-1^; φ(r)=exp[−(εr)^2^] | Numerical interpolation errors not independently re-estimated |
| Brain-shift correction | Local correction with defined anatomical reference | Pre/post displacement and residual error considered separately | No unverified quantitative residual claim |
| Source-cloud size | Patient-level source-cloud inventory | Farthest-point sampling to 512 points | ~227,500 points per patient; see Fig. 4c |

**Table S3.** Comparative performance of DBS outcome prediction models

| **Model** | **Architecture/features** | **Original fixed-split training tolerance rate (%)** | **Original fixed-split held-out tolerance rate (%)** |
| --- | --- | --- | --- |
| Linear regression | 78-D handcrafted feature vector | ≈100 | ≈0 |
| Ridge regression | 78-D handcrafted feature vector; L2 regularization | ≈29 | ≈21 |
| Lasso regression | 78-D handcrafted feature vector; L1 regularization | ≈31 | ≈32 |
| Support vector regression | RBF kernel | ≈69 | ≈32 |
| Gradient boosting decision trees | 78-D handcrafted feature vector | ≈100 | ≈27 |
| Multilayer perceptron | Three hidden layers | ≈26 | ≈21 |
| Voxelized CNN | Regular-grid representation of spatial inputs | — | 33.3 |
| PointNet++ | Hybrid MSG–SSG set abstraction (512→128→32) | — | 62.5 |

Tolerance rate denotes the percentage of continuous predictions within ±5 MDS-UPDRS-III points. Approximate feature-engineered values correspond to the original fixed-split displays; voxelized-CNN and PointNet++ values are 8/24 (33.3%) and 15/24 (62.5%), respectively. The Wilson 95% confidence interval for 15/24 is 42.7%–78.8%.

**Table S4.** Ablation analysis of PointNet++ modules

| **Ablated component** | **Original held-out tolerance rate** | **Decrease from full model (percentage points)** |
| --- | --- | --- |
| Full model (control) | 15/24 (62.5%) | — |
| Remove electric field vectors | 10/24 (41.7%) | 20.8 |
| Remove electrode geometry | 9/24 (37.5%) | 25.0 |
| Remove STN/GPi anatomical labels | 8/24 (33.3%) | 29.2 |
| Replace hierarchical sampling with random | 7/24 (29.2%) | 33.3 |

**Table S5.** Composition of the 78-dimensional feature vector

| **Feature Category** | **Subcategory** | **Specific features** | **Dimensionality** |
| --- | --- | --- | --- |
| Clinical indicators | Demographics | Age, gender (binary), disease duration (years), levodopa equivalent daily dose (LEDD, mg) | 4 |
|  | Preoperative clinical scores | MDS-UPDRS-III total score, tremor subscore, rigidity subscore, bradykinesia subscore | 4 |
|  | Medication history | Levodopa responsiveness (% improvement), prior neurosurgical history (binary) | 2 |
| Imaging features | Anatomical volumetrics | STN volume (mm^3^), GPi volume (mm^3^), putamen volume (mm^3^), thalamus volume (mm^3^) | 4 |
|  | Electrode localization | Electrode-to-STN centroid distance (mm), contact depth relative to STN (mm), electrode trajectory angle (°), and active-contact MNI *x, y,* and *z* coordinates | 6 |
|  | Structural morphology | Cortical thickness (frontal/parietal/occipital lobes), ventricle-to-brain ratio, white matter hyperintensity volume | 5 |
|  | Segmentation metrics | STN surface area (mm^2^), STN eccentricity (sphericity), nucleus boundary irregularity index | 3 |
| Electric field parameters | Magnitude metrics | Mean, median, standard deviation, peak, and 90th-percentile electric-field strength (V/m) | 5 |
|  | Spatial distribution | Field volume (mm^3^) above 1 V/m, field-gradient variance (V/m/mm), isotropic/anisotropic field ratio, field-centroid distance to STN, field-centroid distance to GPi, and field anisotropy index | 6 |
|  | Directional vectors | Mean and variance of azimuth, mean and variance of elevation, and mean *x*- and *y*-direction cosines of electric-field vectors | 6 |
| Biophysical interactions | — | Electrode contact surface area (mm^2^), tissue conductivity (S/m) at the contact site, and contact-to-insulator surface ratio | 3 |
| Derived composite features | Statistical aggregates | Mean, median, maximum, and minimum summaries of selected anatomical and electric-field variables across three spatial summaries: left-sided, right-sided, and pooled bilateral representations | 30 |

**Table S6.** Key software and dependencies for PointNet++ implementation

| **Category** | **Software/Library** | **Version** | **Role** |
| --- | --- | --- | --- |
| Deep learning framework | PyTorch | 1.12.0 | Core framework for model architecture, training, and backpropagation |
| Point cloud processing | PyTorch geometric | 2.1.0 | Tools for point cloud data loading, set abstraction layers, and graph-based operations |
| Neuroimaging analysis | Lead-DBS toolbox | 3.0 | Segmentation of deep-brain nuclei (STN, GPi) from preoperative MRI |
| Biophysical simulation | COMSOL multiphysics | 5.6 | Finite-element modeling of 3D electric field vectors |
| Image registration | Advanced normalization tools (ANTs) | 2.3.4 | Co-registration of preoperative MRI and postoperative CT scans |
| Electrode reconstruction | PaCER algorithm | 1.2 | 3D reconstruction of electrode trajectories and active contact coordinates |
| Numerical computing | NumPy | 1.21.6 | Array operations for point cloud data preprocessing |
| Statistical analysis | SciPy | 1.7.3 | Statistical tests and optimization for model validation |
| Visualization | Matplotlib | 3.5.2 | Visualization of point clouds, electric field distributions, and model performance metrics |

**Table S7.** PointNet++ training, architecture, and validation specifications

| **Component** | **Specification** |
| --- | --- |
| Modeling unit | One independent patient; one selected stimulation–outcome record |
| Original split | 55 training; 24 internal held-out test |
| Repeated nested CV | Not performed for the reported fixed-split analysis |
| Optimizer | Adam |
| Initial/minimum learning rate | 1×10^−4^/1×10^−6^ |
| Scheduler | Cosine annealing |
| Adam parameters | β_1_=0.9; β_2_=0.999; ε=1×10^−8^ |
| Weight decay | 1×10−5 |
| Gradient-norm clip | 10 |
| Batch size | 8 |
| Maximum epochs | 200 |
| Early stopping | Checkpoint selection confined to training-derived data; exact patience not independently verified |
| Checkpoint selection | Training-derived validation criterion only |
| Loss | L_total_=L_reg_+0.1L_corr_; δ=5 |
| Sampling sequence | 512→128→32 points |
| Neighbourhood | Radius-based MSG; separate sample caps by scale |
| MSG radii and samples/radius | Dimensionless radii: layer 1 [0.05,0.1,0.2], layer 2 [0.1,0.2,0.4]; sample caps [16,32,64] in each layer |
| Input channels | *x, y, z*, tissue identity, E_x_, E_y_, E_z_ |
| Augmentation | Rotation ±15°; elastic σ=0.5 mm; point dropout 5%–10% |
| Vector transformation during augmentation | E_x_, E_y_, E_z_ rotated with coordinates using same rotation matrix |
| Dropout probabilities | 0.4 in fully connected layers |
| Random seeds | Python=42, NumPy=2024, PyTorch=42 |
| Trainable parameter count | ~3.6 million |

**Table S8.** Working baseline preprocessing and hyperparameter configurations for the original fixed-split analysis

| **Pipeline component** | **Search space or procedure** | **Selected setting** |
| --- | --- | --- |
| Missing-data handling | Median imputation, fitted on training set only | Median imputation |
| Scaling/normalization | Z-score standardization (mean=0, std=1), fitted on training set only | Z-score standardization |
| Linear regression | fit_intercept=True | fit_intercept=True |
| Ridge regression | α ∈ {0.001, 0.01, 0.1, 1, 10} | α=1.0 |
| Lasso regression | α ∈ {0.001, 0.01, 0.1, 1, 10}, max_iter=10000 | α=0.1 |
| SVR | kernel='rbf', C ∈ {0.1, 1, 10, 100}, γ ∈ {0.001, 0.01, 0.1, 1}, ε=0.1 | C=10, γ=0.1, ε=0.1 |
| GBDT | learning_rate ∈ {0.01, 0.05, 0.1, 0.2}, max_depth ∈ {3,5,7,9} | learning_rate=0.1, max_depth=5 |
| MLP | hidden_layer_sizes ∈ {(64,32), (128,64), (256,128)}, learning_rate ∈ {0.001, 0.01}, α ∈ {0.0001, 0.001} | hidden_layer_sizes=(128,64), learning_rate=0.001, α=0.0001 |
| Voxelized CNN | grid resolution = 2 mm, architecture: Conv3D(1→32→64→128), FC(1024→512→1), optimizer=Adam (lr=1e-3) | grid resolution=2 mm, architecture as specified |
| PointNet++ | Fixed a priori (see Table S7) | See Table S7 |
